# Lack of the brain-specific isoform of apoptosis-inducing factor aggravates cerebral damage in a model of neonatal hypoxia–ischemia

**DOI:** 10.1038/s41419-018-1250-1

**Published:** 2018-12-18

**Authors:** Juan Rodriguez, Yaodong Zhang, Tao Li, Cuicui Xie, Yanyan Sun, Yiran Xu, Kai Zhou, Kaiming Huo, Yafeng Wang, Xiaoyang Wang, Daniel Andersson, Anders Ståhlberg, Qinghe Xing, Carina Mallard, Henrik Hagberg, Nazanine Modjtahedi, Guido Kroemer, Klas Blomgren, Changlian Zhu

**Affiliations:** 1 Center for Brain Repair and Rehabilitation, Institute of Neuroscience and Physiology, Sahlgrenska Academy, University of Gothenburg, Gothenburg, 40530 Sweden; 2 Department of Pediatrics, Children’s Hospital of Zhengzhou University, Zhengzhou, China; 3 Henan Key Laboratory of Child Brain Injury, Third Affiliated Hospital of Zhengzhou University, Zhengzhou, 450052 China; 4 Department of Women’s and Children’s Health, Karolinska Institutet, Stockholm, Sweden; 5 Department of Pediatrics, Second Affiliated Hospital of Hainan Medical University, Haikou, China; 6 Institute of Neuroscience and Physiology, Sahlgrenska Academy, University of Gothenburg, Gothenburg, 40530 Sweden; 7 Sahlgrenska Cancer Center, Department of Pathology and Genetics, Institute of Biomedicine, Sahlgrenska Academy, University of Gothenburg, Gothenburg, 40530 Sweden; 8 Wallenberg Centre for Molecular and Translational Medicine, University of Gothenburg, Gothenburg, Sweden; 9 Institute of Biomedical Science of Fudan University, Shanghai, 201102 China; 10 Center for Perinatal Medicine and Health, Sahlgrenska Academy, University of Gothenburg, Gothenburg, Sweden; 11 INSERM unit U1030, Gustave Roussy Cancer Campus, 94805 Villejuif, France; 12 Metabolomics and Cell Biology Platforms, GRCC, Villejuif, France; Equipe 11 labellisée par la Ligue Nationale contre le Cancer, Centre de Recherche des Cordeliers, INSERM U1138 Paris, France; 13 Université Paris Descartes, Sorbonne Paris Cité, Paris, France; 14 Pôle de Biologie, Hôpital Européen Georges Pompidou, Assistance Publique-Hôpitaux de Paris, Labex Immuno-Oncology, Paris, France; 15 Pediatric Hematology and Oncology, Karolinska University Hospital, Stockholm, Sweden

## Abstract

Apoptosis-inducing factor (AIF) may contribute to neuronal cell death, and its influence is particularly prominent in the immature brain after hypoxia–ischemia (HI). A brain-specific AIF splice-isoform (AIF2) has recently been discovered, but has not yet been characterized at the genetic level. The aim of this study was to determine the functional and regulatory profile of AIF2 under physiological conditions and after HI in mice. We generated AIF2 knockout (KO) mice by removing the AIF2-specific exon and found that the relative expression of *Aif1* mRNA increased in *Aif2* KO mice and that this increase became even more pronounced as *Aif2* KO mice aged compared to their wild-type (WT) littermates. Mitochondrial morphology and function, reproductive function, and behavior showed no differences between WT and *Aif2* KO mice. However, lack of AIF2 enhanced brain injury in neonatal mice after HI compared to WT controls, and this effect was linked to increased oxidative stress but not to caspase-dependent or -independent apoptosis pathways. These results indicate that AIF2 deficiency exacerbates free radical production and HI-induced neonatal brain injury.

## Introduction

Hypoxic–ischemic encephalopathy (HIE) is one of the major causes of neurologic disabilities in neonates. The incidence of HIE ranges from 1 to 8 per 1000 live births in developed countries, increasing to 26 per 1000 live births in underdeveloped countries^1^. If left untreated, moderate to severe HIE implies a 50–65% risk of death or major neurodevelopmental disability, including mental retardation, cerebral palsy, and epilepsy^2^. Despite the recent widespread implementation of hypothermia and erythropoietin therapy, which reduce this risk by approximately 50%^3,4^, HIE remains a significant problem. Therefore, there is an urgent need to explore its pathophysiology and to develop novel strategies for its prevention and treatment.

Neuronal cell death after HI in the developing brain may occur through different modalities including apoptosis, necrosis, necroptosis, ferroptosis, and autophagy^5–8^. Apoptosis is an essential process in the developing brain whereby nerve cells are discretely removed without interfering with the development of remaining cells^9,10^. This can partially explain why many of the key elements of apoptosis, including caspase-dependent and caspase-independent effectors, are strongly upregulated in the immature brain^6,11^.

Apoptosis-inducing factor (AIF) is a key mediator of caspase-independent cell death^12,13^. Beyond its role in lethal processes, AIF has a vital role in oxidative phosphorylation and the detoxification of reactive oxygen species under physiological conditions. AIF depletion causes a deficiency in respiratory chain complex-I activity and decreased ATP production, meaning that AIF plays important roles in cell survival, proliferation, and differentiation by participating in mitochondrial metabolism^14–18^. We previously described that the caspase-independent AIF pathway is involved in perinatal brain injury^11,17^, correlating with the fact that AIF plays a causal role in neuronal cell death and brain injury^19,20^ through its physical interaction with cyclophilin A, generating a protein complex that participates in chromatin degradation^21^. Alternative utilization of exon 2a or 2b of the *Aif* gene can give rise to ubiquitously expressed AIF1 protein (which is commonly referred to as AIF and uses exon 2a) or the brain-specific isoform AIF2 (which uses exon 2b)^22^. Contrasting with AIF1, AIF2 possesses the particularity that it is strongly anchored to the inner mitochondrial membrane (IMM), likely because the stretch of amino acids encoded by exon 2b is rather hydrophobic and yields a domain that is resistant to protease cleavage^22^, while AIF1 can be cleaved by calpains or cathepsins to liberate it from its anchorage in the IMM^23^. For this reason, AIF2 cannot be released from mitochondria and engage in extramitochondrial lethal signaling pathways.

**Fig. 1:**
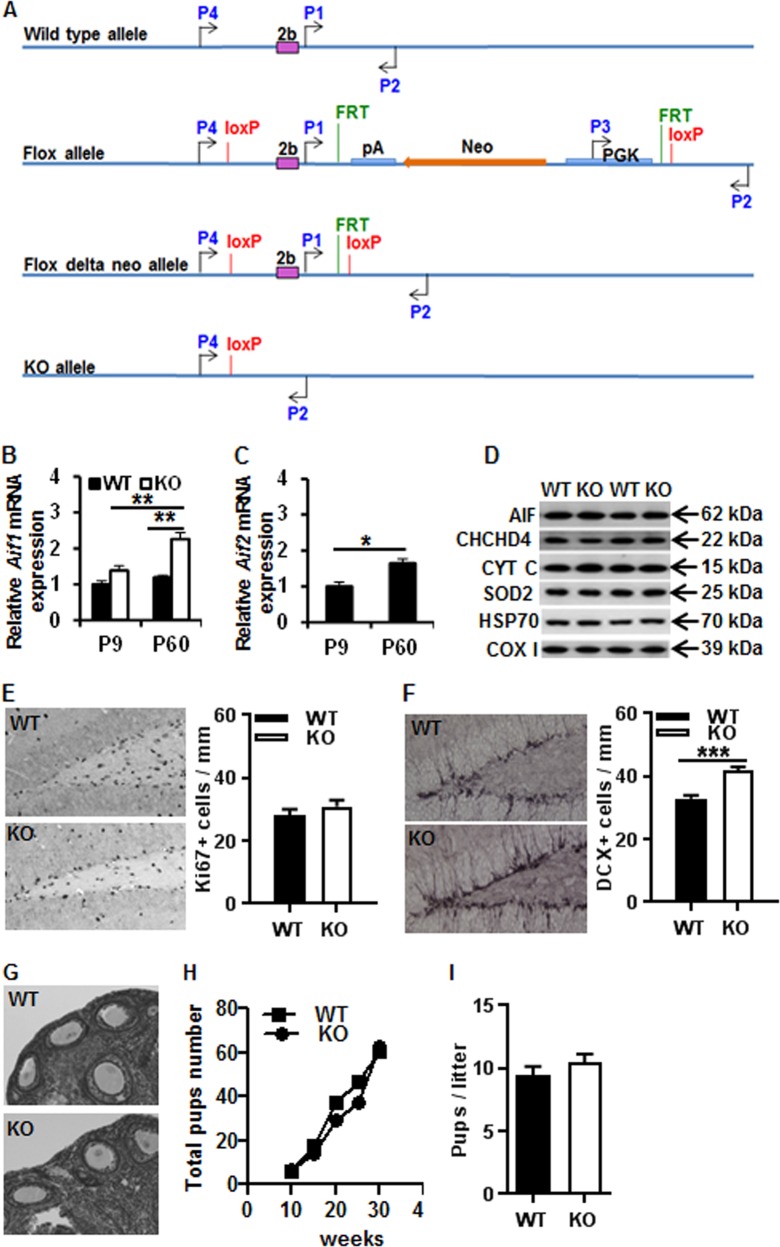
Generation of conditional AIF2 KO mice. **a** The *Aif2* genome locus (WT allele) was modified in embryonic stem cells using the targeting vector. LoxP recombination sites (red bars) along with an FRT (green bar)-flanked neomycin-selection cassette were introduced flanking exon 2b (flox allele). The expression of FLP recombinase results in the removal of the neomycin cassette (flox delta-neo allele). Expression of the Cre recombinase resulted in the excision of exon 2b (KO Allele) and a frameshift, generating a null allele. FRT flippase recognition target sites, FLP flippase. Primer pair P1/P2 distinguishes WT from flox delta neo; primer pair P3/P2 identifies the flox allele; primer pair P4/P2 distinguishes WT from KO. **b** The relative mRNA expression showed that there was a significant increase in *Aif1* mRNA when the *Aif2* mRNA was knocked out in P60 adult mice compared to WT littermates (1.19 ± 0.07 in P9 vs. 2.26 ± 0.17 in P60 *Aif2* KO; ^**^p < 0.01) (P9 WT: *n* = 7; P9 *Aif2* KO: *n* = 8; P60 WT: *n* = 4; P60 *Aif2* KO: *n* = 10). **c** There was a significant increase in the relative expression of *Aif2* mRNA in WT mice at P9 and P60 (1.00 ± 0.12 in P9 vs. 1.64 ± 0.13 in P60; ^*^p < 0.05) (P9: *n* = 7; P60: *n* = 4). **d** Representative western blot analysis of AIF, CHCHD4, cytochrome C (CYT C), SOD2, HSP70, and complex-I 39 kDda subunit (COX I) in both WT and KO mice at P9 under physiological conditions. **e** Representative Ki67 immunostaining in the dentate gyrus of the hippocampus at P60 and quantification of Ki67-positive cells showing no difference between WT and KO. **f** DCX staining in the dentate gyrus. The number of DCX-positive cells was 27% higher in the KO mice (*n* = 8) compared to WT mice (*n* = 13). **g** The photomicrographs show representative ovarian follicles for both WT and KO mice at P60 (*n* = 4 for WT and *n* = 6 for KO). **h** The total number of pups over time, starting from 10 weeks, for WT and KO mice. **i** The average number of pups per litter in WT (litters = 5) and KO mice (litters = 4)

Numerous functional studies have been performed on AIF1^15,17,20,21,24^, whereas AIF2 has not been characterized in detail. The aim of this study was to determine the functional profile of the brain-specific isoform AIF2 under physiological and pathological conditions by means of a knockout (KO) strategy yielding AIF2-deficient mice (Fig. 1a).

## Results

### *Aif2* KO mice have a normal phenotype

The relative abundance of *Aif1* and *Aif2* mRNA transcripts in the brains from the wild-type (WT) control and conditional *Aif2* KO mice littermates aged between postnatal day (P)9 and P60 was determined by reverse transcription quantitative polymerase chain reaction (PCR) (Fig. 1b). Under physiological conditions, the *Aif1* mRNA expression level in conditional *Aif2* KO mice was 38% higher than in WT mice at P9, and this difference was more pronounced as mice aged, reaching a level of 90% higher *Aif1* mRNA expression in *Aif2* KO at 60 days compared to WT (*p* < 0.01) (Fig. 1b). At this age, the levels of *Aif2* mRNA were also higher than at P9 in WT mice (Fig. 1c). However, the mitochondria-related proteins, including total AIF (AIF1 + AIF2), CHCHD4, cytochrome C, SOD2, HSP70, and complex-I subunit, showed no difference between WT and *Aif2* KO mouse pups in the brain at P9 (Fig. 1d). To examine the impact of AIF2 on the neural stem/progenitor cell proliferation condition, Ki67 was analyzed at P60 in both WT and *Aif2* KO mice (Fig. 1e), and no difference was observed in the number of Ki67-labeled cells between WT and *Aif2* KO mice. The neurogenesis condition, as detected by using doublecortin (DCX) immunostaining, showed that the density of DCX-labeled cells was 27% higher in *Aif*2 KO mice compared to WT mice at P60, suggesting that the *Aif2* KO favors neurogenesis (Fig. 1f).

To investigate the influence of *Aif2* KO on fertility, follicular development in *Aif2* KO ovaries was assayed. Follicles at various developmental stages ranging from primordial to pre-ovulatory were found in *Aif2* KO ovaries at P60, which was comparable to WT ovaries. This shows that *Aif2* KO does not affect normal follicle development (Fig. 1g). We also checked the sexual maturation time of female *Aif2* KO mice and observed vaginal opening at the age of 5–6 weeks, which was similar to WT control mice. To determine whether *Aif2* KO influences fertility, we housed *Aif2* KO female mice with WT males. We found that the fertility of *Aif2* KO females was comparable to that of WT females during the testing period from 8 weeks to 16 weeks of age, indicating that *Aif2* KO did not affect the fertility of female mice (Fig. 1h). Furthermore, the number of pups in each litter was similar for WT and *Aif2* KO mice (Fig. 1i).

**Fig. 2:**
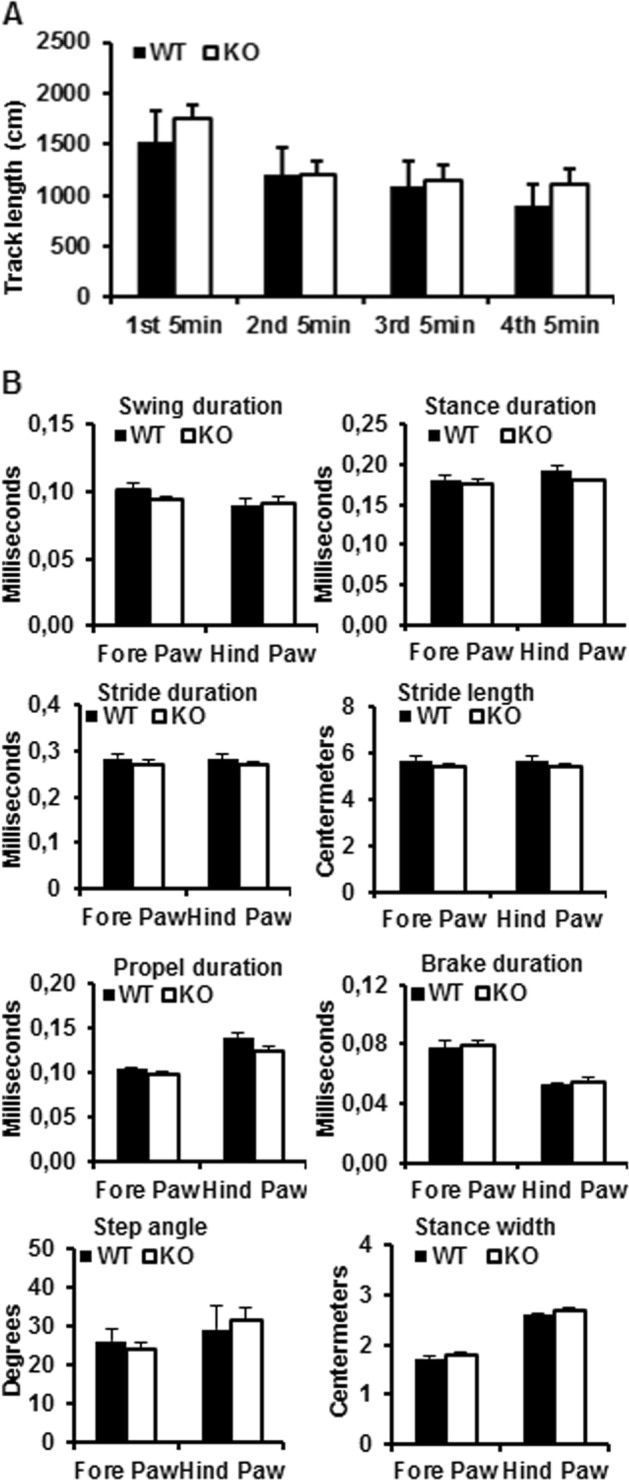
Behavioral tests. **a** Open field results showing the running distance in each 5 min interval for a total of 20 min. **b** Gait analysis profile, including swing duration, stance duration, stride duration, stride length, propel duration, brake duration, step angle, and stance width. There were no differences in gait profile between WT and AIF2 KO mice. *n* = 11 for WT, and *n* = 5 for AIF2 KO

At 18 months of age there were no visible phenotypic differences between *Aif2* KO and WT mice, which exhibited a similar level of survival (data not shown). To examine if the brain isoform of AIF2 affects neural functional development, a battery of neurobehavioral tests were performed, including open field (Fig. 2a) and DigiGate (Fig. 2b). Again, we found no obvious differences in learning and memory, running activity, or motor function between WT and *Aif2* KO mice.

**Fig. 3:**
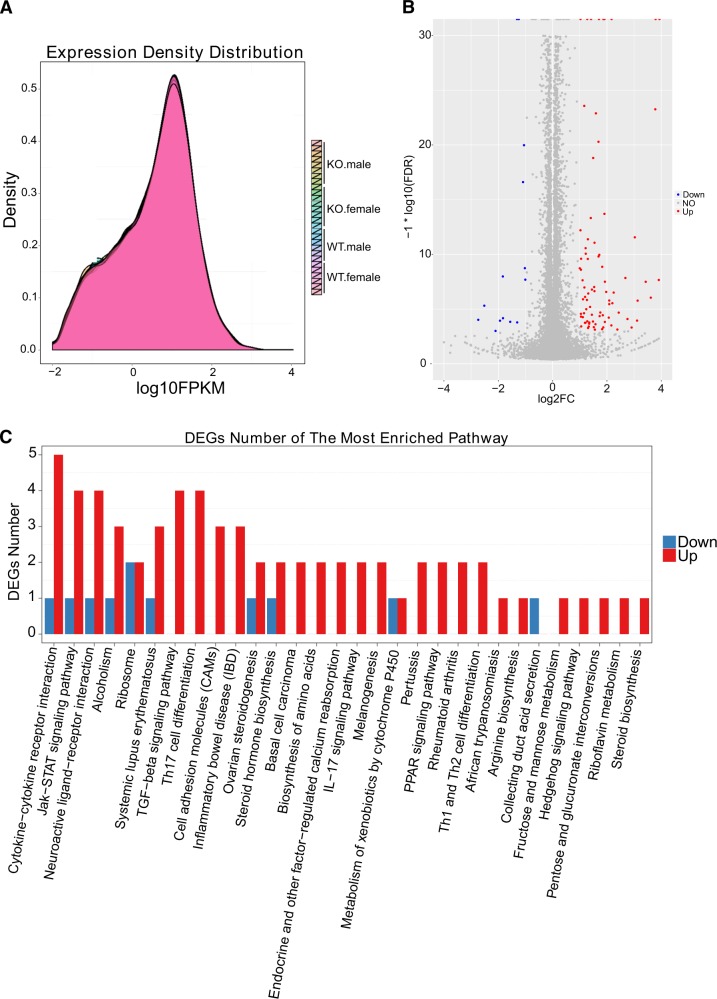
AIF2 KO has no significant impact on the transcriptome in the cortex of P9 mice. **a** The density map shows no difference in the abundance or concentration of gene expression between the WT and KO groups. **b** Scatter plots of all expressed genes in each pairwise RNA sequence analysis in the cortex of P9 mice. **c** KEGG pathway enrichment analyses of all significantly altered genes from the cortex. Pathways that are reduced or increased are shown in blue and red, respectively. **c** No single pathway was enriched by more than five differentially expressed genes (DEGs). The filter criteria were established as a fold change > 2 and *q* < 0.01. *n* = 12 for WT (6 males and 6 females), and *n* = 16 for AIF2 KO (8 males and 8 females)

To understand the potential impact of *Aif*2 KO in the cortex of the mouse brain under physiological conditions, the transcriptome changes were assayed at P9 by whole transcriptome sequencing in both *Aif*2 KO and WT mice. The data showed no major difference regarding to the abundance and concentration of total gene expression in the cortex between the two genotypes (Fig. 3a), while of the 21,827 genes assayed, 109 genes were upregulated in *Aif*2 KO mice and 15 genes were downregulated compared to the WT mice (Fig. 3b). However, KEGG pathway enrichment analyses did not reveal any coherent changes in specific pathways (Fig. 3c).

### Impact of *Aif2* KO on mitochondrial function

**Fig. 4:**
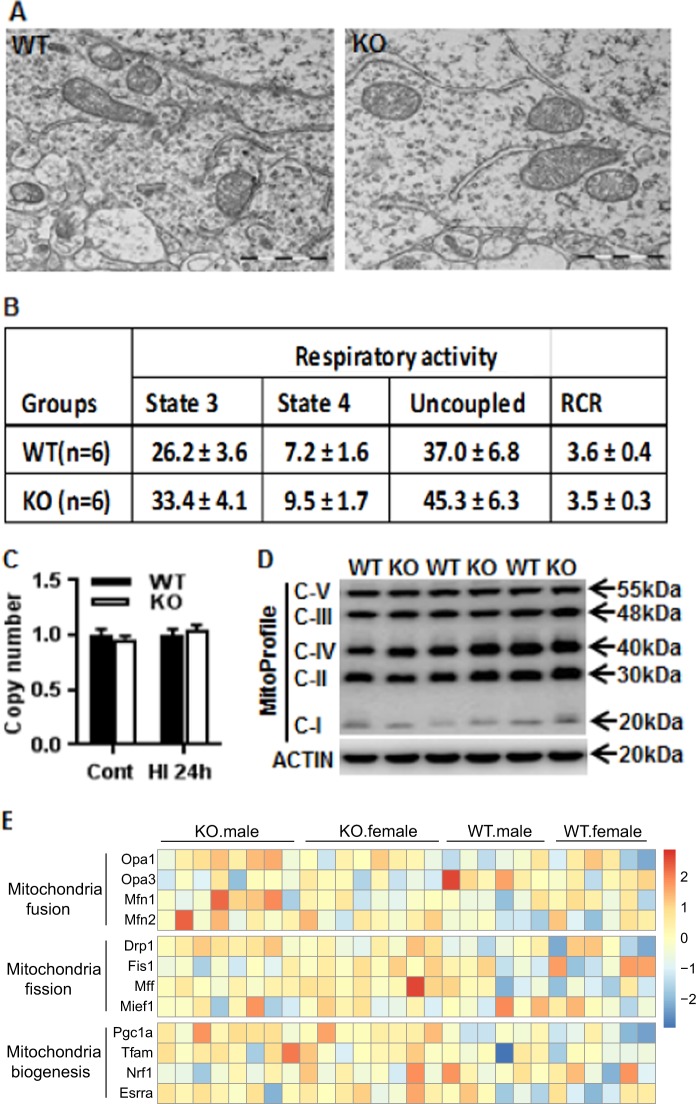
AIF2 KO does not affect mitochondrial morphology or respiratory activity. **a** Representative photomicrographs in a cortical neuron show the morphology and number of mitochondria in WT and AIF2 KO mice at P9. There were no obvious differences between the two genotypes in terms of mitochondrial morphology (*n* = 2 for WT, *n* = 4 for AIF2 KO). **b** Mitochondrial respiratory activity in the WT and AIF2 KO mice at P9. There were no significant differences in the average values of respiratory activities or the respiratory control ratio (RCR). **c** The mtDNA copy number was not significantly different between WT and KO mouse pups under physiological conditions or at 24 h after HI (*n* = 6/group). **d** Immunoblotting of respiratory chain complexes I–V subunits in the mitochondrial fraction of WT and KO mice at P9 (*n* = 6/group). **e** Transcriptome results showed no significant changes in FPKM of mitochondrial fusion, fission, or biogenesis genes between the WT and KO group under physiological conditions. *n* = 6 for WT males, *n* = 6 for WT females, *n* = 8 for AIF2 KO males, and *n* = 8 for AIF2 KO females

Mitochondrial morphology and distribution in cortical neurons were investigated by electron microscopy, and these showed no differences between P9 WT and *Aif2* KO pups (Fig. 4a). Mitochondrial respiratory activity, as indicated by the respiratory control ratio (state 3 respiration/state 4 respiration), was not significantly different between WT and *Aif2* KO mouse pups at P9 (Fig. 4b). We also checked mitochondrial biogenesis by mitochondrial DNA (mtDNA) copy number assay (Fig. 4c) and mitochondrial respiratory chain complex subunit expression in the mitochondrial fraction (Fig. 4d), again not detecting any tangible impact of the *Aif2* KO on mitochondrial biogenesis at P9. This was further confirmed by a mitochondrial biogenesis-related gene assay. The expression of mitochondrial fusion and fission-related proteins was not influenced by the absence of AIF2 (Fig. 4e).

### *Aif2* KO increases brain injury in neonatal HI mice

**Fig. 5:**
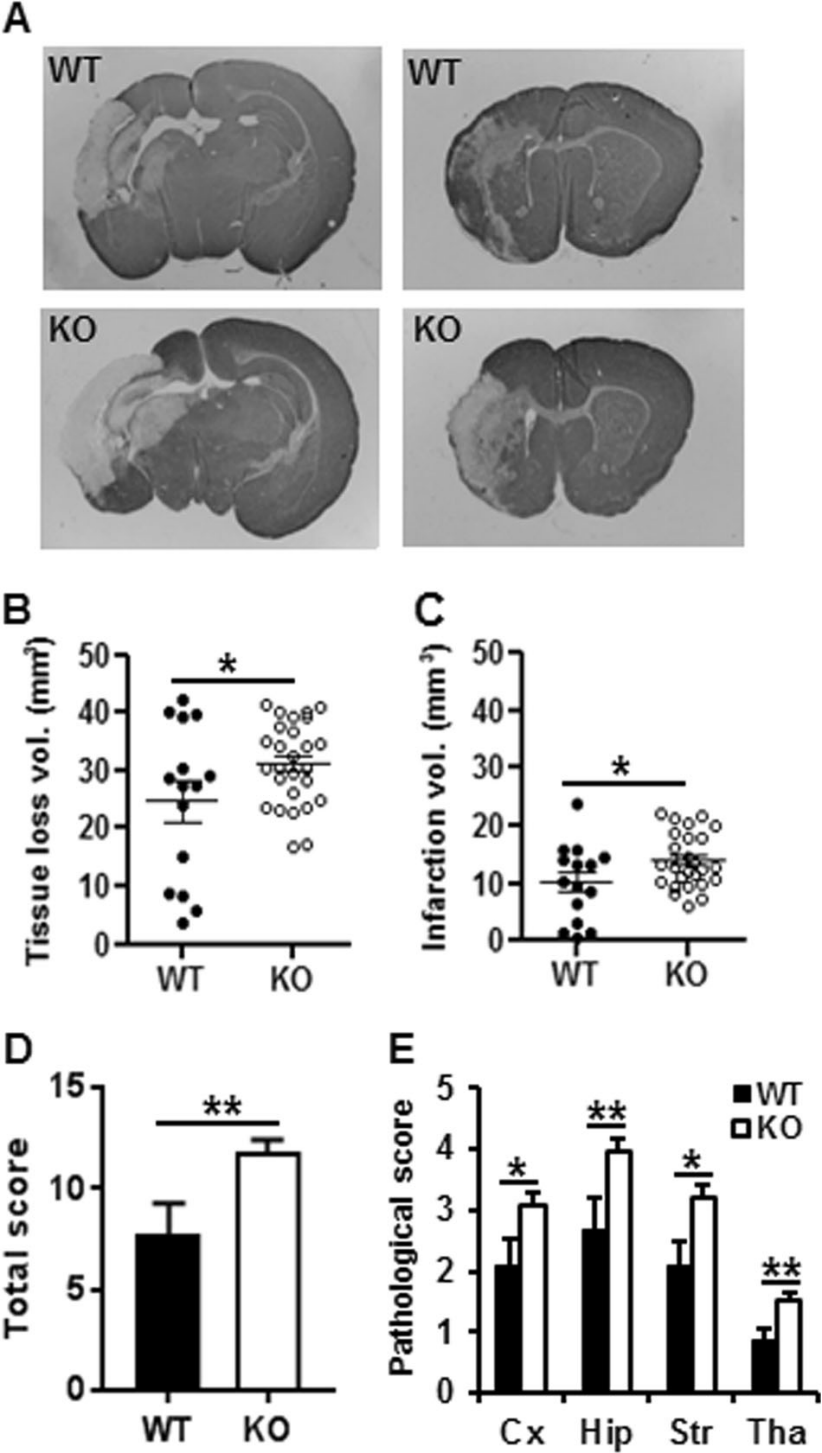
Knockout of AIF2 increases brain injury in neonatal mice after HI. **a** The photomicrographs show representative MAP2 staining at 72 h after HI at the dorsal hippocampus (left panel) and the striatum (right panel) levels in WT and KO mice. **b** The total tissue loss volume at 72 h after HI (24.7 ± 3.4 mm^3^ for WT, *n* = 15 vs. 31.1 ± 1.4 mm^3^ for AIF2 KO, *n* = 27, ^*^p < 0.05). **c** The infarction volume at 72 h after HI (10.1 ± 1.7, *n* = 15 vs. 13.9 ± 0.9, *n* = 27, ^*^p < 0.05). **d** The total pathological score (7.72 ± 1.54, *n* = 15 vs. 11.77 ± 0.64, *n* = 27, ^*^p < 0.05). **e** The pathological scores in the cortex (2.13 ± 0.45 vs. 3.09 ± 0.20, ^*^p < 0.05), hippocampus (2.66 ± 0.52 vs. 3.97 ± 0.21, ^*^p < 0.05), striatum (2.07 ± 0.42 vs. 3.20 ± 0.22, ^*^p < 0.05), and thalamus (0.86 ± 0.20 vs. 1.51 ± 0.13, ^*^p < 0.05) in WT and KO mice at 72 h post-HI

HI-induced brain injury in neonatal mice was evaluated by MAP2 immunohistochemistry staining as a marker of gray matter at 72 h post-HI. The injury encompassed the cortex, hippocampus, striatum, and thalamus as indicated by the MAP2 staining on brain coronal sections (Fig. 5a). Of note, the volume of tissue loss was greater in *Aif2* KO mice compared to WT mice (*p* < 0.05) (Fig. 5b). Thus, the infarction volume corresponding to the MAP2-negative areas was increased in *Aif2* KO mice compared to their WT littermates (*p* < 0.05) (Fig. 5c). The total neuropathological score was significantly higher for *Aif2* KO mice compared to WT controls (*p* < 0.01) (Fig. 5d), and this difference was especially pronounced in the hippocampus (*p* < 0.01), striatum (*p* < 0.05), and thalamus (*p* < 0.01), but also detectable at the level of the cortex (*p* < 0.05) (Fig. 5e).

### Impact of *Aif2* KO on apoptotic cell death

**Fig. 6:**
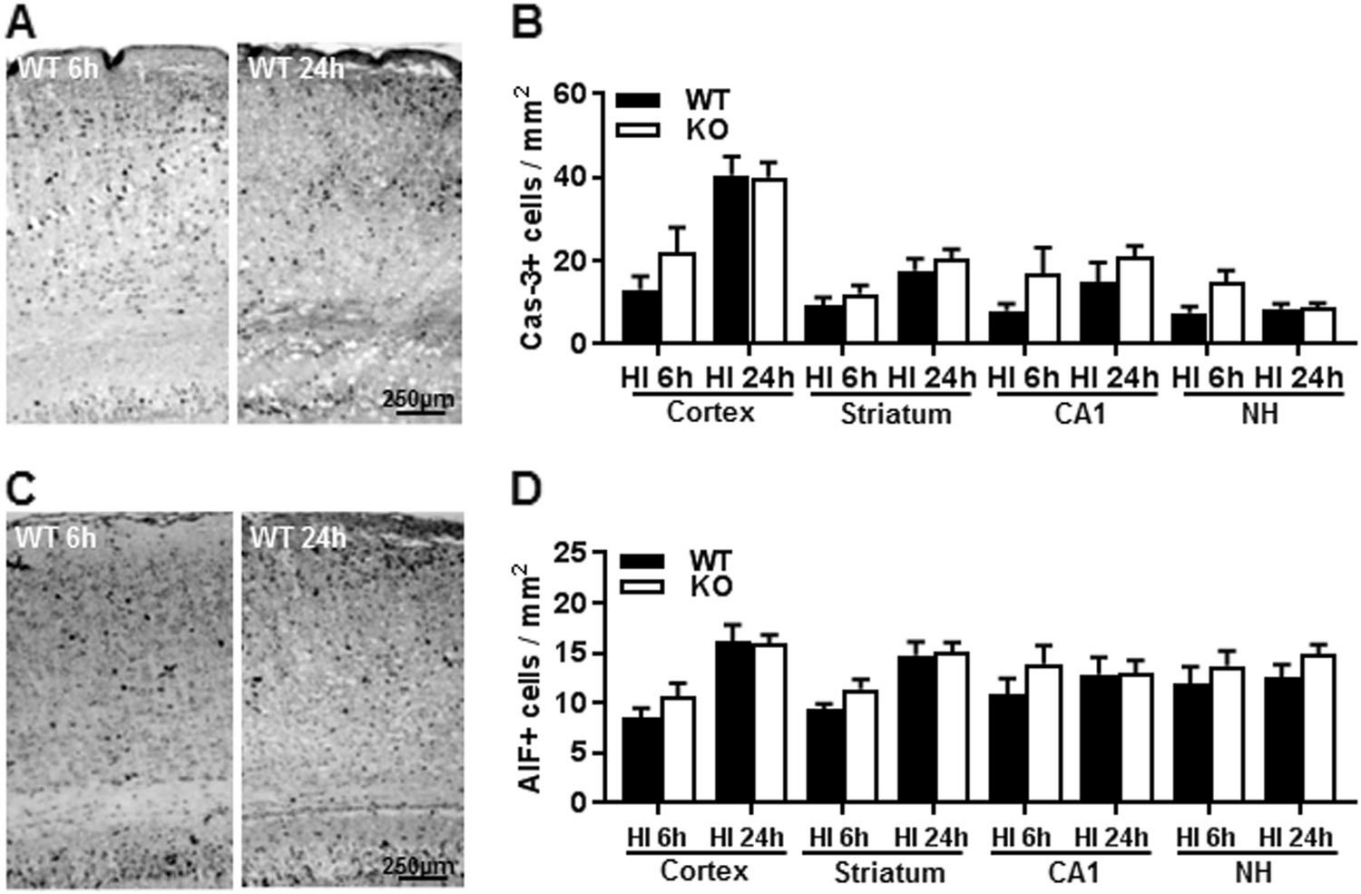
AIF2 KO has no effect on caspase-dependent and independent apoptosis. **a** Representative images show staining for the active form of caspase-3 at 6 and 24 h after HI. **b** Quantification of the number of active caspase-3-positive cells in WT and KO mice at 6 and 24 h post-HI. The bar graph has been divided into four sets for the cortex (CX), striatum (Str), CA1, and nuclei habenula (NH). There were no significant differences in the numbers of active caspase-3-positive cells. **c** Representative images showing the staining for the active form of AIF at 6 and 24 h after HI. **d** Quantification of the AIF-positive cells (nuclear AIF immunostaining) in WT and KO at 6 and 24 h post-HI. There were no significant differences between WT and KO at 6 h (*n* = 9/group) or 24 h (n = 8/group) after HI

Active caspase-3-labeled cells in different brain regions were measured at 6 and 24 h post-HI (Fig. 6a). The frequencies of active caspase-3-labeled cells increased significantly in the cortex at 24 h compared to 6 h in both groups of mice, and a similar tendency was seen in the striatum and CA1 area of the hippocampus. In the nucleus habenularis (NH), active caspase-3-labeled cells reached their peak at 6 h. Although there was a tendency for more active caspase-3-positive cells in the *Aif2* KO group at 6 h after HI, this difference did not reach statistical significance in all the examined brain regions (Fig. 6b). We then checked caspase-independent AIF-mediated apoptosis in different brain regions at 6 and 24 h post-HI (Fig. 6c). AIF-positive nuclei were quantified, and the results showed no difference between WT and *Aif2* KO mice at 6 or 24 h after HI (Fig. 6d).

### AIF2 KO induces oxidative stress after HI

**Fig. 7:**
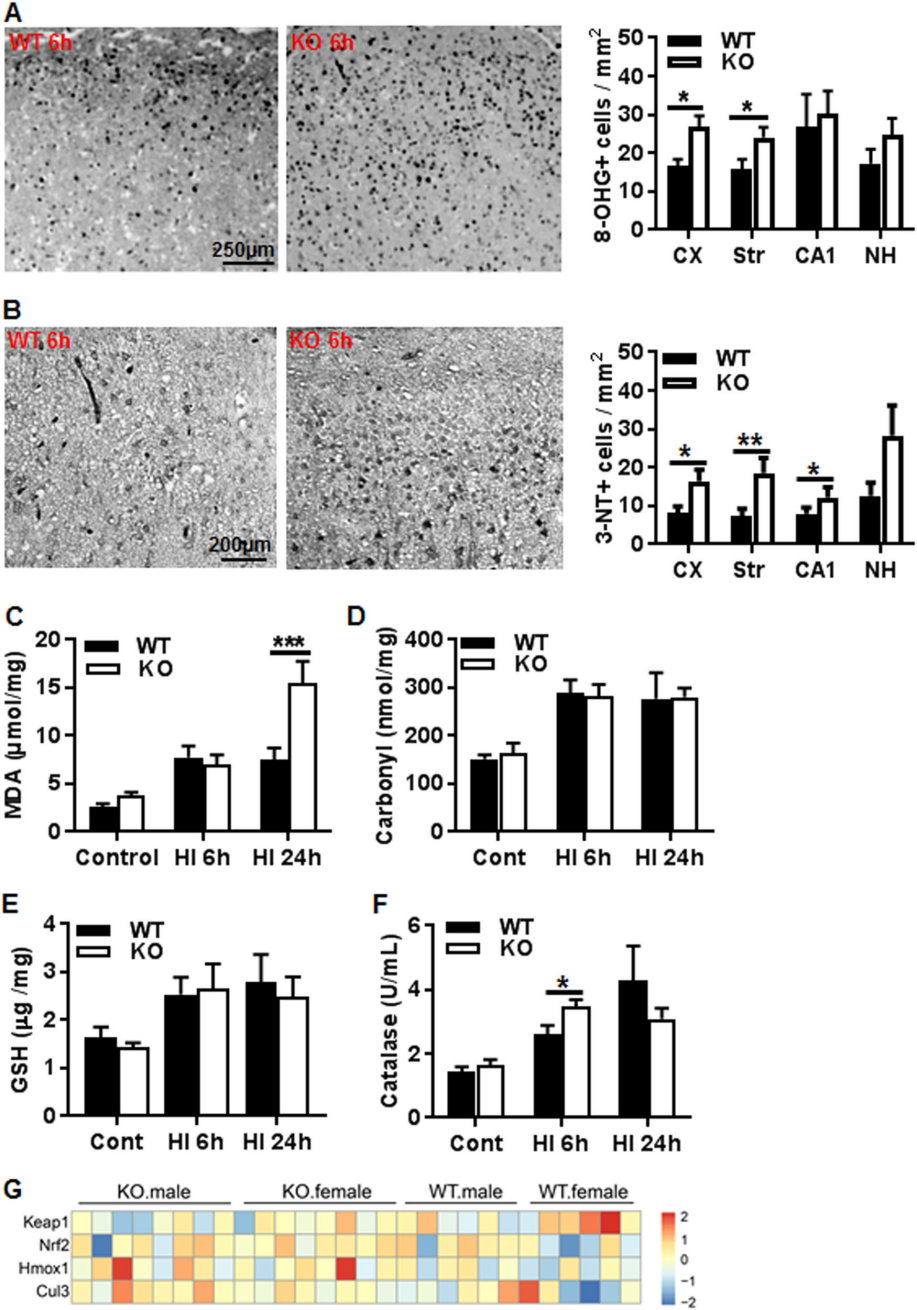
AIF2 KO induces oxidative stress. **a** Representative images of 8-OHG immunostaining in the cortex of WT and KO mice at 6 h after HI. The bar graph shows quantification of the 8-OHG-labeled cells in the cortex (Cx), striatum (Str), hippocampal cornus ammonis 1 area (CA1), and nucleus habenularis (NH) in WT and KO mice at 6 h after HI. *n* = 6 for WT and *n* = 7 for AIF2 KO. **b** Representative images of 3-NT immunostaining in cortex of WT and KO at 6 h after HI. The bar graph shows quantification of 3-NT-positive cells in different brain regions in WT and KO mice at 6 h after HI. *n* = 6 for WT and *n* = 7 for AIF2 KO. **c** Quantification of malondialdehyde (MDA) in WT and KO mice under physiological conditions (Cont) and at 6 and 24 h post-HI. At 24 h after HI, there was a significant increase in KO mice compared to WT littermates (7.53 ± 1.22 vs. 15.44 ± 2.31, ^***^p < 0.001) *n* = 5 for WT control and *n* = 7 for KO control; n = 6 for WT and KO at 6 h post-HI; *n* = 5 for WT at 24 h post-HI and *n* = 9 for KO at 24 h post-HI. **d** Quantification of protein carbonylation in homogenates from WT and KO mice under physiological conditions (Cont) and at 6 and 24 h post-HI. There was a significant increase of carbonylated protein after HI, but no difference between WT and KO. *n* = 4 for WT control and *n* = 5 for KO control; *n* = 6 for WT and KO at 6 h post-HI; *n* = 5 for WT at 24 h post-HI and *n* = 9 for KO at 24 h post-HI. **e** Quantification of glutathione (GSH) in WT and KO mice under physiological conditions (control) and at 6 and 24 h post-HI. There were no significant differences between WT and KO mice (*n* = 6/group). **f** Quantification of catalase activity in cortical mitochondrial fraction from WT and KO mice under physiological conditions and at 6 and 24 h post-HI. The activity was increased after HI. It was 25% higher at 6 h and 28% lower in KO compared to WT mice. *n* = 6 for WT and KO control; *n* = 4 for WT and *n* = 7 KO at 6 h post-HI; *n* = 5 for WT at 24 h post-HI and *n* = 7 for KO at 24 h post-HI. **g**. The heat map shows the FPKM of cellular redox capacity-related genes, and no significant differences were detected between the WT and KO groups under physiological conditions. *n* = 6 for WT males, *n* = 6 for WT females, *n* = 8 for AIF2 KO males, and *n* = 8 for AIF2 KO females

8-Hydroxyguanosine (8-OHG) is a marker of oxidative damage to nucleic acids. Cells with 8-OHG-positive nuclei were counted in different brain regions at 6 h after HI. The frequency of 8-OHG-positive nuclei was significantly higher in the cortex (61%) (*p* < 0.05) and striatum (53%) (*p* < 0.05) from *Aif2* KO mice compared to WT mice (Fig. 7a). We then determined protein nitrosylation by staining with antibodies recognizing 3-nitrotyrosine (3-NT), a stable marker of peroxynitrite formation, in different brain regions at 6 h after HI. The number of 3-NT-positive cells was significantly higher in the cortex, striatum, and CA1 of the hippocampus from Aif2 KO mice as compared to WT mice (Fig. 7b). We then measured malondialdehyde (MDA) and protein carbonyl content in cortical tissues. MDA is one of the final intracellular products of polyunsaturated fatty acid peroxidation, and hence commonly considered as a marker of oxidative stress. There were no significant differences in MDA levels between WT and *Aif2* KO mice under physiological condition or at 6 h post-HI. However, there was a dramatic increase (105%) at 24 h in the *Aif2* KO mice compared to WT mice (*p* < 0.001) (Fig. 7c). Protein carbonylation is another marker of oxidative stress. The quantity of carbonyls in in the cortical homogenate samples increased significantly after HI, but there was no difference between WT and *Aif2* KO mice under physiological conditions or after HI (Fig. 7d). Moreover, glutathione (GSH) levels in cortical brain tissue under normal conditions and at 6 and 24 h post-HI were similar for WT and *Aif2* KO mice (Fig.7e). We also measured the catalase activity contained in cortical mitochondrial fractions. HI insult stimulated catalase activation in both WT and *Aif2* KO mice, and this activity tended to be lower at 24 h after HI in *Aif2* KO mice compared to WT mice, though without reaching statistical significance (Fig. 7f). We also checked the expression of proteins involved in the Keap1-Nrf2 pathway, which is the major regulator of cytoprotective responses to oxidative stress, finding no significant change between WT and *Aif2* KO mice at baseline or at P9 (Fig. 7g). These genes were double checked by reverse transcription quantitative PCR, supporting similar expression levels in WT and KO mice (data not shown).

## Discussion

AIF is considered to be a major contributor to neuron loss induced by cerebral ischemia and trauma in both neonates and adults, because these conditions cause the release of AIF from mitochondria and its translocation to nuclei, where it is detected immediately after insult, only in damaged areas^19,20,25,26^. Harlequin mice, which have an 80% reduction in the expression of both AIF isoforms (AIF1 and AIF2)^22^, exhibit a significant reduction in brain injury. Thus, male and female Harlequin pups demonstrate a 52.6 and 41% reduction in infarct volume at 72 h post-HI^19^. Similarly, adult Harlequin mice show a 43% reduction at 24 h upon focal cerebral ischemia as compared to WT controls^25^. However, the brain-specific isoform AIF2 has not been studied in the context of neuronal cell death. We initially hypothesized that AIF2 might contribute to brain damage. However, we found that KO of AIF2 actually increased HI-induced brain injury.

Under physiological conditions, both AIF1 and AIF2 are integrated in the IMM where they contribute to the biogenesis of complex-I of the mitochondrial respiratory chain and might catalyze redox reactions as well^22^. The two AIF isoforms only differ in their N-terminal region^22^. This segment of AIF is used to anchor the protein in the IMM, and upon apoptotic stimuli it is cleaved off by calpains and cathepsins, causing the rest of the protein to be released from the IMM into the mitochondrial intermembrane space and, if the outer mitochondrial membrane is permeabilized, into the cytosol, allowing for its translocation to the nucleus^15,22^. It has been hypothesized that the AIF2 brain-specific isoform is more tightly anchored in the lipid bilayer due to the differences in the amino acid sequence of the N-terminal region. This segment might be important for differences in AIF’s accessibility to apoptotic proteases and thus might regulate its release from the mitochondria into the cytosol^27^. It has also been suggested that AIF1 and AIF2 might be able to interact with each other to form homodimers or heterodimers^22^. If this is the case, AIF2 might be neuroprotective by “kidnaping” AIF1 molecules at the IMM, thus blocking their pro-apoptotic function.

The results presented here also revealed that the relative expression of *Aif1* was significantly higher in *Aif2* KO adult mice. The levels of *Aif2* increase with aging, suggesting that the increased expression of *Aif1* might be a compensatory reaction to the absence of *Aif2*. Our results indicate a noticeable increase (by 38%) in *Aif1* mRNA in P9 *Aif2* KO mice compared to age-matched WT littermates, and this increase was even more pronounced (90%) for adult (P60) *Aif2* KO mice. However, we could not quantify the protein levels of the two AIF isoforms because there are no isoform-specific antibodies that would allow us to discriminate between them. Furthermore, cell death mechanisms after ischemic insult in the brain are developmentally regulated, and fundamental differences exist between the responses of the immature and the adult brain to HI^6,28^. Caspase-3 activity and the numbers of caspase-3-positive cells were similar for both WT and *Aif2* KO mice, in line with the caspase-independency of the lethal pathway mediated by AIF^29^.

Mitochondria not only function in metabolism cell death regulation, but also coordinate inflammation and the repair of cellular injury^14,30,31^. In the course of neuronal death signaling, pro-apoptotic proteins such as AIF are released from mitochondria to the cytosol and then translocate to the nucleus, where they contribute to lethal processes such as chromatinolysis. Inhibition of the mitochondrio-nuclear translocation of AIF and the reduction of AIF expression both reduce neuronal cell death and brain injury^21,32^. Cyclophilin A and AIF form a protein complex that contributes to nuclear DNA degradation, and inhibiting the interaction between cyclophilin A and AIF protects against oxidative stress-induced neuronal cell death^32^. CHCHD4 is a mitochondrial protein that physically interacts with AIF^16^, and AIF depletion or deletion is coupled to decreased CHCHD4 protein expression without *CHCHD4* mRNA levels are affected^16,18^. We recently demonstrated that haploinsufficiency of CHCHD4 (induced by heterozygous KO of the gene) reduces neuronal cell death and caspase-independent apoptotic cell death^24^. However, *Aif2* KO did not affect the expression of CHCHD4 protein, perhaps due to the compensatory upregulation of AIF1.

Previous studies revealed the potential effect of AIF on neuronal differentiation^22,33^. Even though no phenotype is associated with *Aif2* KO mice, DCX-positive cells in the subgranular zone (SGZ) of the dentate gyrus of the hippocampus were more numerous in P60 *Aif2* KO mice than in age-matched WT controls, suggesting that *Aif2* KO induces neurogenesis by affecting physiological neural developmental processes in adult mice. It should be noted that adult Harlequin mutant mice, with about an 80% reduction in AIF expression (both AIF1 and AIF2), exhibit a progressive loss of adult cerebellar and retinal neurons. Because AIF2 is only expressed in the brain, it was hypothesized that this specific isoform might be crucial for neurogenesis^22^. However, here we found that *Aif2* KO mice were not only viable, but that neurogenesis was stimulated under physiological conditions. The reasons why an increased expression of AIF1 (and lack of AIF2) promotes neurogenesis remain elusive. These will be studied in the future using an AIF1 overexpression mouse model that we are currently building.

Studies using Harlequin mutant mice suggested that AIF serves as a free radical scavenger because this mouse model exhibits increased amounts of hydroperoxides in the brain^19,34^. Thus, AIF downregulation increased oxidative stress, which is an early feature after HI, and the immature brain is especially vulnerable to free radicals^6,35^. Mitochondria, where AIF is located, play crucial roles in the activation of apoptotic mechanisms and serve as both a source and as a target of free radicals, and this feature might be affected by the local presence of AIF, which plays an important role in mitochondrial metabolism^36,37^. The abundance of markers of oxidative stress, including 8-OHG, 3-NT, and MDA increased in *Aif2* KO mice after HI insult without significant changes in GSH levels or the Keap1-Nrf2 pathway. This suggests that local redox equilibria are affected by the lack of AIF2 or the overabundance of AIF1. However, the interaction between AIF2 and redox reactions needs to be investigated in further detail.

In summary, our results suggest that there is probably a compensatory effect in the expression level of AIF1 when AIF2 is knocked out and that this change in the AIF1/AIF2 ratio favors increased oxidative stress and subsequent brain injury after HI. However, this needs further confirmation in an adult HI model. We anticipate that the in-depth understanding of this process might lead to the development of effective treatments for the early stages of perinatal brain injury.

## Materials and methods

### AIF2 mouse breeding and genotyping

Conditional AIF2 KO mice were generated (Ozgene company, Australia) by inserting *loxP* sites flanking exon 2b of the *Aif* gene (Fig. 1a). *Aif2*^flox/flox^ mice were crossed with a homozygous PGK-Cre-driven line (the transgene directs the expression of Cre recombinase under the control of a phosphoglycerate kinase promoter)^38^ to produce brain *Aif2*^flox/flox.PGK-Cre^ KO (*Aif2* KO) and *Aif2*^flox/+.PGK-Cre^ control mice (WT). Genomic DNA was isolated from tail samples according to the manufacturer’s instructions (Qiagen, Hilden, Germany; 69506). PCR was performed on an Applied Biosystems GeneAmp19700 PCR system by using four different primer pairs: primer pair P1/P2 5′-TTG GGG ATA GGG TGG GTA AGA C-3′ (forward) and 5′-GAG GTA ACA AAA GGG ACA CTG CC-3′ (reverse), which allowed the amplification of a 518 base pair (bp) fragment from the WT allele, a 2377 bp from the flox allele, and a 637 bp fragment from the flox delta-neo allele; Primer pair P3/P2: 5′-GAG GTA ACA AAA GGG ACA CTG CC-3′ (forward) and 5′-GAA AGC GAA GGA GCA AAG CTG-3′ (reverse), which allowed the amplification of a 836 bp fragment from the flox allele; Primer pair P4/P2 5′-GAG AAC AAA AGG TTG GAT GCC AC-3′ (forward) and 5′-GAA AGC GAA GGA GCA AAG CTG-3′ (reverse), which allowed the amplification of a 1062 bp fragment from the WT allele and a 568 bp fragment from the KO allele; and 5′-GCA AGC TTT CGA CCA TGC CCA AGA AGA AG-3′ (forward) and 5′-GCG AAT TCC GTT AAT GGC TAA TCG CCA TC-3′ (reverse), which allowed the amplification of a 380 bp fragment from the PGK-cre allele. PCR products were separated on a 1.5% agarose gel containing SYBR Green. A 100 bp ladder was used to verify the sizes of the PCR products. The gels were imaged with an LAS 3000 cooled CCD camera (Fujifilm, Tokyo, Japan).

### Hypoxia–ischemia

Postnatal day 9 (P9) mice of both sexes were anesthetized with isoflurane (5% for induction, 1.5% to 2.0% for maintenance) in a 1:1 mixture of nitrous oxide and oxygen, and the duration of anesthesia was less than 5 min. The left common carotid artery was cut between double ligatures of Prolene sutures (6.0). After the surgical procedure, the wounds were infiltrated with lidocaine for local analgesia. The pups were returned to their cages for 1 h and then placed in a chamber perfused with a humidified gas mixture (10% oxygen in nitrogen) for 50 min at 36 °C. Following hypoxic exposure, the pups were returned to their cages. Control pups were subjected to all procedures except HI. Mice were maintained in the Laboratory for Experimental Biomedicine of Gothenburg University^17^. All experiments were performed in accordance with international guidelines and approved by the Gothenburg Committee of the Swedish Animal Welfare Agency (Application nos. 111/2014 and 112/2014).

### Immunohistochemical staining

At 6 or 24 h after HI-induced brain injury, the pups were deeply anesthetized with 50 mg/ml phenobarbital and perfused intracardially with PBS and 5% buffered formaldehyde (Histofix; Histolab, Gothenburg, Sweden). Their brains were removed and fixed in 5% buffered formaldehyde at 4 °C for 18–24 h. After dehydration with graded ethanol and xylene, the brains were paraffin-embedded and cut into 5 μm coronal sections. Every 100th section throughout the whole brain was stained for microtubule-associated protein 2 (MAP2), and every 50th section in the hippocampus was stained for active caspase-3, AIF, 3-NT, and 8-OHG. Briefly, sections were deparaffinized in xylene and rehydrated in graded ethanol concentrations. Antigen retrieval was performed by heating the sections in 10 mM boiling sodium citrate buffer (pH 6.0) for 10 min. Nonspecific binding was blocked for 30 min with 4% donkey or goat serum in PBS for 30 min. The primary antibodies were monoclonal mouse anti-MAP2 (1:1000 dilution, clone HM-2, Sigma, M9942), rabbit anti-active caspase-3 (1:150 dilution, BD Pharmingen, 559565), goat anti-AIF (1:100 dilution, 2 μg/ml, Santa Cruz Biotechnologies, sc-9416), mouse anti-3-NT (1:200, ab53232, Abcam), and mouse anti–8-OHG (1:200, ab48508, Abcam). After incubating the sections with the primary antibodies at 20 °C for 60 min or overnight at 4 °C, the appropriate biotinylated secondary antibodies (1:200 dilutions; all from Vector Laboratories, Burlingame, CA, USA) were added for 60 min at room temperature. After blocking endogenous peroxidase activity with 3% H_2_O_2_ for 10 min, the sections were visualized with Vectastain ABC Elite (Vector Laboratories) and 0.5 mg/mL 3,3,9-diaminobenzidine enhanced with ammonium nickel sulfate, β-d glucose, ammonium chloride, and β-glucose oxidase. After dehydrating with graded ethanol and xylene, the sections were mounted using Vector mounting medium.

### Brain-injury evaluation

The gray-matter area was determined by measuring the MAP2 immunoreactive area from eight serial sections per animal. Brain injury was evaluated by the volume of infarction and total hemispheric tissue loss and neuropathological scoring, which was performed by a person who did not have prior knowledge of the groups. The MAP2-positive and negative areas in each section were measured in both hemispheres using Micro Image (Olympus, Japan). The tissue volume was calculated from the MAP2-positive or negative areas according to the Cavalieri principle using the following formula: *V* = Σ*A* × *P* × *T*, where *V* = the total volume, Σ*A* = the sum of area measurements, *P* = the inverse of the sampling fraction, and *T* = the section thickness. The total hemispheric tissue loss was calculated as the MAP2-positive volume in the contralateral hemisphere minus the MAP2-positive volume in the ipsilateral hemisphere. The neuropathological score of gray matter from different brain regions was assessed. Briefly, the cortical injury was graded from 0 to 4 with 0 being no observable injury and 4 indicating confluent infarction. The injury in the hippocampus, striatum, and thalamus was assessed both with respect to hypotrophy (scored from 0 to 3) and injury/infarction (scored from 0 to 3), resulting in a total possible score of 22^24^.

### Cell counting

Area contours were drawn and measured in every 50th section. The active caspase-3-positive and AIF-positive nuclei and the 8-OHG-positive cells were counted at ×400 magnification in the border zone of the injured cortex and striatum, cornu ammonis area 1 (CA1), and NH within an area of 0.196 mm^2^ (one visual field) using Micro Image (Olympus, Japan). Three sections were counted from each brain with an interval of 250 μm^39^. The numbers of Ki-67-positive and DCX-positive cells were counted in the SGZ of the dentate gyrus in three adjacent sections in the hippocampus. The length of the SGZ was measured, and the number of positive cells was expressed as the number per mm. The average was defined as *n* = 1 when comparing different brains. All of the counting was carried out by investigators blinded to group assignment.

### Sample preparation for immunoblotting and colorimetric assay

The pups were sacrificed by decapitation. Tissue from the cortex (including the hippocampus) in each hemisphere was rapidly dissected out on a bed of ice and homogenized immediately using a 2 mL glass/glass homogenizer (Merck Eurolab, Gothenburg, Sweden) on ice, and isolation buffer was added (15 mM Tris-HCl (pH 7.6), 320 mM sucrose, 1 mM dithiothreitol, 3 mM EDTA-K, and 0.5% protease inhibitor cocktail, which was added fresh immediately prior to use). Half of the homogenates were aliquoted and stored at −80 °C, and the other half were centrifuged at 800×*g* at 4 °C for 10 min. The pellet was washed, recentrifuged, and saved as the nuclear fraction. The supernatants were further centrifuged at 9200×*g* for 15 min at 4 °C, producing mitochondrial and synaptosomal fractions in the pellet and crude cytosolic fractions in the supernatants. The enriched mitochondrial fraction was washed and centrifuged. All fractions were stored at −80 °C.

### Immunoblotting

A total of 65 μl of each mitochondrial fraction sample was mixed with 25 μl NuPAGE LDS 4× sample buffer and 10 μl reducing agent and then heated at 70 °C for 10 min. Individual samples were run on 4–12% NuPAGE Bis-Tris gels (Novex, San Diego, CA, USA) and transferred to reinforced nitrocellulose membranes (Schleicher & Schuell, Dassel, Germany). After blocking with 30 mM Tris-HCl (pH 7.5), 100 mM NaCl, and 0.1% Tween 20 (TBS-T) containing 5% fat-free milk powder for 1 h at room temperature, the membranes were incubated with anti-AIF (sc-9416, 1:1000 dilution, 0.2 μg/ml, goat polyclonal antibody, Santa Cruz Biotechnology, Dallas, TX, USA), anti-cytochrome c (clone 7H8.2C12, 1:500, Pharmingen, San Diego, CA, USA), anti-actin (1:200, rabbit polyclonal antibody, Sigma), anti-OxPhos Complex-I 39 kDa subunit (clone 20C11, 1:1000, 0.5 μg/ml, Molecular Probes, Eugene, OR, USA), total oxidative phosphorylation system rodent western blot antibody cocktail (MS604, 1:250, MitoSciences, Eugene, OR, USA), anti-superoxide dismutase II (Mn-SOD/SOD2, clone 2A1, 1:1000, 0.5 μg/ml, Lab Frontier, Seoul, Korea), anti-HSP70 (sc-7298, 1:500, mouse monoclonal antibody, Santa Cruz Biotechnology) or anti-CHCHD4 (sc-98628, 1:500, rabbit polyclonal antibody, Santa Cruz, CA, USA) primary antibodies at room temperature for 60 min. After washing, the membranes were incubated with a peroxidase-labeled secondary antibody for 30 min at room temperature (goat anti-rabbit 1:2000, horse anti-goat 1:2000, or horse anti-mouse 1:4000). Immunoreactive species were visualized using the Super Signal West Dura substrate (Pierce, Rockford, IL, USA) and an LAS 3000 cooled CCD camera (Fujifilm).

### Oxidative stress assay

The levels of MDA (TBARS assay kit, Cayman Chemical), protein carbonylation (Abcam, ab126287, Cambridge, UK), GSH (R&D System, Minneapolis, MN, USA), and catalase expression (Thermo Fisher Scientific Inc. EIACATC, Carlsbad, CA, USA) in the ischemic and control brains were measured with commercially available kits according to the manufacturers’ instructions. The absorbance was read on a microplate reader (Molecular Devices Corp., Sunnyvale, CA, USA).

### RNA extraction and sequencing

In total, 28 cortical samples from P9 WT and AIF2 KO mice were prepared for RNA sequencing. Total RNA from each sample was extracted using an RNeasy Mini kit (Qiagen), and the library preparation was done using an MGI Easy^™^ mRNA Library Prep Kit (BGI, Inc., Wuhan, China.) following the manufacturer’s instructions. The sequencing library was used for cluster generation and sequencing on the BGISEQ-500 system (BGI, Inc.)^40^. The sequencing was repeated ten times, and the DESeq method was used to screen for differentially expressed genes between the two groups according to the criteria of a fold change > 2 and *q* < 0.01.

### Real-time quantitative PCR

For gene-expression profiling of Aif1/Aif2, RNA was reversed transcribed using TATAA GrandScript cDNA Synthesis Kit (TATAA Biocenter, Gothenburg, Sweden) in single 10 μl reactions containing 500 ng total RNA, according to manufacturer’s instructions. All cDNA samples were diluted with nuclease-free water to a total volume of 100 μl. Quantitative real-time RT-qPCR was performed with a CFX384 Touch real-time cycler (Bio-Rad, Hercules, CA, USA). Each 6 μl reaction contained 1× TATAA SYBR GrandMaster Mix (TATAA Biocenter, Gothenburg, Sweden), 400 nM of each primer, and 2 μl cDNA. The temperature profile was 95 °C for 2 min followed by 40 cycles of amplification (95 °C for 5 s, 60 °C for 20 s and 70 °C for 20 s). All samples were analyzed by melting curve analysis. Relative quantification using reference genes was applied. Twelve potential reference genes from the Mouse Reference Gene Panel (TATAA Biocenter, Gothenburg, Sweden) were evaluated with NormFinder; *Actb* and *Pgk1* were chosen as optimal reference genes. The following primers used in the RT-qPCR reactions were designed by Beacon Designer software (free trial, PREMIER Biosoft): common forward primer for *Aif1* and *Aif2*, 5′-CGA GCC CGT GGT ATT CGA-3′, *Aif1* reverse, 5′-CCA TTG CTG GAA CAA GTT GC-3′; and *Aif2* reverse, 5′-CTA GGA GAT GAC ACT GCA CAA-3′. To confirm mitochondria biogenesis, fusion and fission, as well as oxidative stress-related genes, RT-qPCR was done as we described previously^24,39^.

### Mitochondrial DNA copy number measurement

Total DNA from the cortex was isolated using a genomic DNA isolation kit (DNeasy Blood & Tissue Kit, Qiagen). The amount of mitochondrial DNA relative to nuclear genomic DNA was determined by RT-qPCR. The nuclear gene was *Ywhaz* (sense: 5′-GAG GAA GAA TCG TGA GTT AGT T-3′, anti-sense: 5′-TGG TGA TGG TTG AGA CAG A-3′), and the mitochondrial gene was *ND4* (sense: 5′-CCT CAG TTA GCC ACA TAG C-3′, anti-sense: 5′-GAT TCG TTC GTA GTT GGA GTT-3′). The relative mitochondrial DNA level was calculated based on the threshold cycle (Ct) as 2-Δ (ΔCt)^41^.

### Electron microscopy

The brains were perfused and then fixed in the same solution (2.5% glutaraldehyde, 2% paraformaldehyde, 0.05% sodium azide, and 0.05 M sodium cacodylate) for 24 h, and then transferred to PBS. Whole hemispheres were sliced into 0.5 mm thick coronal sections, and pieces from the parietal cortex were collected. Post-fixing was performed in 1% OsO_4_ at 4 °C for 2 h, and the samples were treated with 0.5% aqueous uranyl acetate for 2 h. The samples were dehydrated and embedded in epoxy resin (Agar 100 Resin). Ultrathin sections (60 nm) were cut on a Reichert Ultramicrotome (Reichert Microscope Services, Depew, NY), post-stained with 5% uranyl acetate, and examined with an LEO 912AB transmission electron microscope (Carl Zeiss NTS GmbH, Oberkochen, Germany) equipped with a Proscan and a Megaview III camera (Olympus Soft Imaging Solutions GmbH, Münster, Germany). Analysis of mitochondrial ultrastructure, size, and distribution in the cortex was performed.

### Ovary histology analysis

Ovaries were fixed in 4% paraformaldehyde, dehydrated, and embedded in paraffin. The paraffin-embedded ovaries were serially sectioned at 8 mm thickness and rehydrated followed by staining with hematoxylin for morphological observation. Ovarian follicles at different developmental stages were categorized based on the well-accepted standards established by Pedersen and Peters.

### DigiGait analysis

DigiGait equipment was purchased from Mouse Specifics, Inc. (Boston, USA). Briefly, each mouse was placed on a motor-driven treadmill with a transparent treadmill belt and imaged from beneath with a high-speed digital video camera. A minimum of three seconds of movie is required for DigiGait analysis, and mice that could not run at the chosen speed for that duration were deemed to have failed to complete the test. Color images were converted to their binary matrix equivalents, and the areas of the moving paws relative to the belt and camera were calculated throughout each stride. This was used to generate a dynamic gait signal of the paw placement relative to the treadmill belt and camera. Each limb′s gait signal was used to calculate the stride duration (the time for one complete stride for the paw under analysis). This was broken down into subcomponents of stance duration (the time during which the paw is in contact with the treadmill) and swing duration (the time during which the paw is above the walking surface and not in contact with the belt). Stance duration further comprises the braking phase (time from initial paw contact with the treadmill to maximum paw contact) and propulsion duration (time from maximum paw contact to lifting from the treadmill). Stride width was defined as the perpendicular distance between the centroids of each set of axial paws during peak stance. Gait symmetry was measured as the ratio of forelimb stepping frequency to hind limb stepping frequency^42^.

### Open field

Open field analysis allows the detection of exploration-related motor activity, habitation, and anxiety-related activity. Briefly, the mice were individually introduced into an unfamiliar 20 cm × 20 cm square arena with 30 cm high walls made of gray polypropylene. The arena was placed in the center of a brightly lit room. Each mouse was released in the center of the arena and allowed to explore for 20 min. The middle of the animal’s body was defined as the point for tracking entries into different zones. The data, including movement distance, activity, and time spent in the central zone, were recorded using the View II software (BIOBSERVE, Germany) and automatically summarized every 5 min. The arena was cleaned with 70% alcohol after each animal was tested^43^.

### Statistical analysis

The Statistical Package for the Social Sciences 21.0 (SPSS, IBM, NY, USA) was used for all analyses. Comparisons between groups were performed by Student’s *t*-test, and one-way ANOVA was used for multiple comparisons (least significant difference and Bonferroni tests) when equal variances between groups were assumed. Data with unequal variance were compared with the Mann–Whitney *U*-test. Results are presented as means ± standard errors of the mean (SEM), and *p* < 0.05 was considered statistically significant.

## References

[1] Douglas-Escobar M, Weiss MD (2015). Hypoxic–ischemic encephalopathy: a review for the clinician. JAMA Pediatr..

[2] Laptook AR (2017). Effect of therapeutic hypothermia initiated after 6h of age on death or disability among newborns with hypoxic-ischemic encephalopathy: a randomized clinical trial. J. Am. Med. Assoc..

[3] Edwards AD (2010). Neurological outcomes at 18 months of age after moderate hypothermia for perinatal hypoxic ischaemic encephalopathy: synthesis and meta-analysis of trial data. Br. Med. J..

[4] Zhu C (2009). Erythropoietin improved neurologic outcomes in newborns with hypoxic–ischemic encephalopathy. Pediatrics.

[5] Northington FJ, Chavez-Valdez R, Martin LJ (2011). Neuronal cell death in neonatal hypoxia–ischemia. Ann. Neurol..

[6] Zhu C (2005). The influence of age on apoptotic and other mechanisms of cell death after cerebral hypoxia–ischemia. Cell Death Differ..

[7] Galluzzi L (2018). Molecular mechanisms of cell death: recommendations of the Nomenclature Committee on Cell Death 2018. Cell Death Differ..

[8] Galluzzi L, Bravo-San Pedro JM, Blomgren K, Kroemer G (2016). Autophagy in acute brain injury. Nat. Rev. Neurosci..

[9] Thornton C (2017). Cell death in the developing brain after hypoxia–ischemia. Front. Cell. Neurosci..

[10] Oppenheim RW (2008). Developing postmitotic mammalian neurons in vivo lacking Apaf-1 undergo programmed cell death by a caspase-independent, nonapoptotic pathway involving autophagy. J. Neurosci..

[11] Zhu C (2003). Involvement of apoptosis‐inducing factor in neuronal death after hypoxia–ischemia in the neonatal rat brain. J. Neurochem..

[12] Susin SA (1999). Molecular characterization of mitochondrial apoptosis-inducing factor. Nature.

[13] Wang Y., et al. A nuclease that mediates cell death induced by DNA damage and poly(ADP-ribose) polymerase-1. *Science* **354**, pii6872 (2016). 10.1126/science.aad6872 PMC5134926 27846469

[14] Hagberg H, Mallard C, Rousset CI, Thornton C (2014). Mitochondria: hub of injury responses in the developing brain. Lancet Neurol..

[15] Hangen E, Blomgren K, Bénit P, Kroemer G, Modjtahedi N (2010). Life with or without AIF. Trends Biochem. Sci..

[16] Hangen E (2015). Interaction between AIF and CHCHD4 regulates respiratory chain biogenesis. Mol. Cell.

[17] Sun Y, Zhang Y, Wang X, Blomgren K, Zhu C (2012). Apoptosis-inducing factor downregulation increased neuronal progenitor, but not stem cell, survival in the neonatal hippocampus after cerebral hypoxia–ischemia. Mol. Neurodegener..

[18] Meyer K (2015). Loss of apoptosis-inducing factor critically affects MIA40 function. Cell Death Dis..

[19] Zhu C (2007). Apoptosis-inducing factor is a major contributor to neuronal loss induced by neonatal cerebral hypoxia-ischemia. Cell Death Differ..

[20] Slemmer JE (2008). Causal role of apoptosis-inducing factor for neuronal cell death following traumatic brain injury. Am. J. Pathol..

[21] Zhu C (2007). Cyclophilin A participates in the nuclear translocation of apoptosis-inducing factor in neurons after cerebral hypoxia–ischemia. J. Exp. Med..

[22] Hangen E (2010). A brain-specific isoform of mitochondrial apoptosis-inducing factor: AIF2. Cell Death Differ..

[23] Otera H, Ohsakaya S, Nagaura Z, Ishihara N, Mihara K (2005). Export of mitochondrial AIF in response to proapoptotic stimuli depends on processing at the intermembrane space. EMBO J..

[24] Sun Y (2017). Haploinsufficiency in the mitochondrial protein CHCHD4 reduces brain injury in a mouse model of neonatal hypoxia-ischemia. Cell Death Dis..

[25] Culmsee C (2005). Apoptosis-inducing factor triggered by poly(ADP-ribose) polymerase and Bid mediates neuronal cell death after oxygen-glucose deprivation and focal cerebral ischemia. J. Neurosci..

[26] Thal SE, Zhu C, Thal SC, Blomgren K, Plesnila N (2011). Role of apoptosis inducing factor (AIF) for hippocampal neuronal cell death following global cerebral ischemia in mice. Neurosci. Lett..

[27] Churbanova IY, Sevrioukova IF (2008). Redox-dependent changes in molecular properties of mitochondrial apoptosis-inducing factor. J. Biol. Chem..

[28] Wang X (2009). Developmental shift of cyclophilin D contribution to hypoxic-ischemic brain injury. J. Neurosci..

[29] Candé C (2002). Apoptosis-inducing factor (AIF): a novel caspase-independent death effector released from mitochondria. Biochimie.

[30] Galluzzi L, Kepp O, Kroemer G (2012). Mitochondria: master regulators of danger signalling. Nat. Rev. Mol. Cell Biol..

[31] Wang X (2010). Neuroprotective effect of Bax-inhibiting peptide on neonatal brain injury. Stroke.

[32] Doti N (2014). Inhibition of the AIF/CypA complex protects against intrinsic death pathways induced by oxidative stress. Cell Death Dis..

[33] Ishimura R, Martin GR, Ackerman SL (2008). Loss of apoptosis-inducing factor results in cell-type-specific neurogenesis defects. J. Neurosci..

[34] Klein JA (2002). The harlequin mouse mutation downregulates apoptosis-inducing factor. Nature.

[35] Wang X (2007). N-acetylcysteine reduces lipopolysaccharide-sensitized hypoxic–ischemic brain injury. Ann. Neurol..

[36] Sun Y (2016). Dichloroacetate treatment improves mitochondrial metabolism and reduces brain injury in neonatal mice. Oncotarget.

[37] Tobaben S (2011). Bid-mediated mitochondrial damage is a key mechanism in glutamate-induced oxidative stress and AIF-dependent cell death in immortalized HT-22 hippocampal neurons. Cell Death Differ..

[38] Lallemand Y, Luria V, Haffner-Krausz R, Lonai P (1998). Maternally expressed PGK-Cre transgene as a tool for early and uniform activation of the Cre site-specific recombinase. Transgenic Res..

[39] Xie C (2016). Neuroprotection by selective neuronal deletion of Atg7 in neonatal brain injury. Autophagy.

[40] Xu Y (2018). Cranial irradiation induces hypothalamic injury and late-onset metabolic disturbances in juvenile female rats. Dev. Neurosci..

[41] Zhou K (2017). Radiation induces progenitor cell death, microglia activation, and blood–brain barrier damage in the juvenile rat cerebellum. Sci. Rep..

[42] Zhang X (2017). gammadeltaT cells but not alphabetaT cells contribute to sepsis-induced white matter injury and motor abnormalities in mice. J. Neuroinflamm..

[43] Xie C, Zhou K, Wang X, Blomgren K, Zhu C (2014). Therapeutic benefits of delayed lithium administration in the neonatal rat after cerebral hypoxia–ischemia. PLoS One.

